# Unexpected Uterine Rupture—A Case Report, Review of the Literature and Clinical Suggestions

**DOI:** 10.3390/jcm12103532

**Published:** 2023-05-18

**Authors:** Wojciech Flis, Maciej W. Socha, Mateusz Wartęga, Rafał Cudnik

**Affiliations:** 1 Department of Perinatology, Gynecology and Gynecologic Oncology, Faculty of Health Sciences, Collegium Medicum in Bydgoszcz, Nicolaus Copernicus University, Łukasiewicza 1, 85-821 Bydgoszcz, Poland; 2 Department of Obstetrics and Gynecology, St. Adalbert’s Hospital in Gdańsk, Copernicus Healthcare Entity, Jana Pawła II 50, 80-462 Gdańsk, Poland; 3 Department of Pathophysiology, Faculty of Pharmacy, Collegium Medicum in Bydgoszcz, Nicolaus Copernicus University, M. Curie-Skłodowskiej 9, 85-094 Bydgoszcz, Poland

**Keywords:** VBAC, TOLAC, uterine rupture, cesarean section, induction of labor

## Abstract

Background and Objectives: Women with a history of cesarean section are a high-risk group because they are likely to develop uterine rupture during their next pregnancy. Current evidence suggests that a vaginal birth after cesarean section (VBAC) is associated with lower maternal mortality and morbidity than elective repeat cesarean delivery (ERCD). Additionally, research suggests that uterine rupture can occur in 0.47% of cases of trial of labor after cesarean section (TOLAC). Case Description: A healthy 32-year-old woman at 41 weeks of gestation, in her fourth pregnancy, was admitted to the hospital due to a dubious CTG record. Following this, the patient gave birth vaginally, underwent a cesarean section, and successfully underwent a VBAC. Due to her advanced gestational age and favorable cervix, the patient qualified for a trial of vaginal labor (TOL). During labor induction, she displayed a pathological CTG pattern and presented symptoms such as abdominal pain and heavy vaginal bleeding. Suspecting a violent uterine rupture, an emergency cesarean section was performed. The presumed diagnosis was confirmed during the procedure—a full-thickness rupture of the pregnant uterus was found. The fetus was delivered without signs of life and successfully resuscitated after 3 min. The newborn girl of weight 3150 g had an Apgar score of 0/6/8/8 at 1, 3, 5, and 10 min. The uterine wall rupture was closed with two layers of sutures. The patient was discharged 4 days after the cesarean section without significant complications, with a healthy newborn girl. Conclusions: Uterine rupture is a rare but severe obstetric emergency and can be associated with maternal and neonatal fatal outcomes. The risk of uterine rupture during a TOLAC attempt should always be considered, even if it is a subsequent TOLAC.

## 1. Introduction

The rupture of the pregnant uterus is an uncommon but highly severe obstetrical event strongly associated with high fetal and maternal morbidity during the peripartum period. A uterine rupture is the complete division of all three layers of the uterine muscle: the endometrium, myometrium, and perimetrium. Most uterine ruptures occur during late gestation. Uterine rupture has become an important topic in recent years due to the increasing percentage of patients being offered a TOLAC. TOLAC refers to a plan to have a vaginal birth in any subsequent pregnancy after cesarean labor. Uterine rupture is by far the most dangerous possible complication that can occur during TOLAC. Typically, uterine rupture occurs suddenly and requires immediate critical emergency care for mothers and neonates. The risk of this life-threatening condition varies from 0.012 to 0.3%, depending on the region. In less developed countries, the risk of uterine rupture is much higher, mainly due to poor obstetric care. There are many risk factors for an intrapartum uterine rupture, with the predominance of a previous cesarean section [1,2,3]. Most commonly, it occurs during a subsequent TOLAC. Research suggests that the risk of uterine rupture may range from 0.47% to approximately 0.87% during TOLAC [4,5]. However, there are also cases of uterine rupture in primigravid patients [6]. 

The rate of cesarean sections (CS) has increased significantly over the past few decades. Available data show that the rate of CS continues to rise globally, now accounting for more than 21% of all childbirths [7].

This paper presents a case of uterine rupture after a second TOLAC attempt, together with a review of the literature and a proposal of clinical suggestions based on this case and clinical experience. 

## 2. Case Description

In her fourth pregnancy, a healthy 32-year-old woman at 40 + 6 weeks of gestation was admitted to the hospital due to a dubious CTG record. She complained of mild lower abdomen pain. The patient’s first child was born by spontaneous delivery. The second pregnancy was ended by CS due to a breech position, followed by the delivery of a healthy child by VBAC two years later. She had no significant medical history, and her antenatal care had been uneventful. During routine CTG recording at hospital admission, the FHR baseline showed normal variability with accelerations. During the recording, there was a single deceleration without systolic activity (up to about 80 beats per minute), which was immediately corrected to normal values. The CTG record was evaluated. No repetitive decelerations were found. The recording was assessed as normal according to the FIGO classification [8]. During admission, the patient was clinically stable. Lab results showed hemoglobin = 10.7 g/dL, white blood cells = 7.72 G/L, platelets = 223 G/L, C-reactive protein = 1.2 mg/dL, and an activated partial thromboplastin time (aPTT) of 24.4 s. Vitals were stable. An ultrasound revealed a eutrophic child in the longitudinal occipitoanterior position, with an estimated fetal weight of 3300 g and a normal amount of amniotic fluid. The biophysical profile test score result (Manning’s score) was 10/10. There were no ultrasonographic signs of abruption of the placenta or placenta previa. Doppler examination of the umbilical and middle cerebral arteries showed a normal spectrum of blood flow. Sonographic measurement of the lower uterine segment (LUS) thickness showed 3.4 mm. There was no bleeding or oozing of amniotic fluid during a vaginal examination. The Bishop score of the cervical examination was >6, with 3–4 cm cervical dilation and 60–70% cervical effacement [9]. During her stay in the pregnancy pathology ward, several repeat CTG recordings were performed, which showed no disturbing symptoms and were classified as normal according to the FIGO classification.

**Figure 1:**
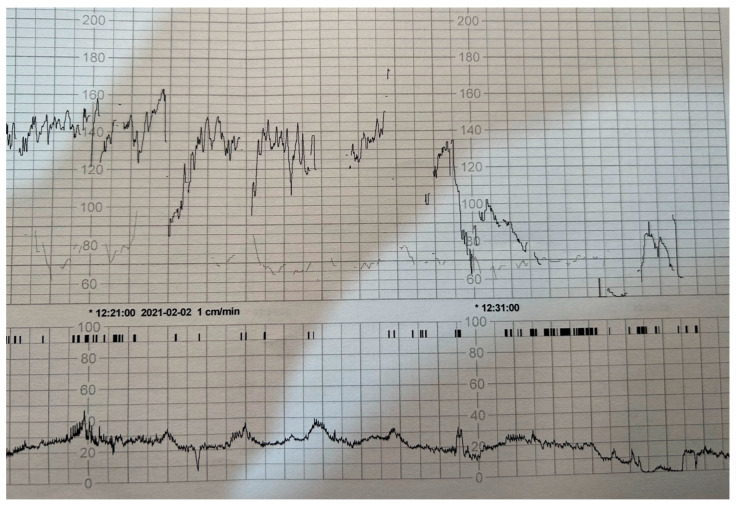
CTG recording showing prolonged deceleration; upper black line—fetal heart rate record; lower black line—uterine systolic activity; gray line—maternal heart rate record.

Using the VBAC calculator [10,11], the chances of a successful vaginal birth were estimated at 95%. Due to her advanced gestational age and favorable cervix (Bishop > 6), the patient qualified for a trial of vaginal labor (TOL). According to the local induction of labor (IOL) low-dose protocol, an oxytocin infusion containing 5 units in 19 milliliters of a saline solution was set up. Induction of labor was started with an initial dose of 0.5 mUI/mL, increasing the dose by 1 mUI/min. After an hour and a half, the patient developed strong contractions. The labor pain was relieved using Entonox gas (a mixture of 50% nitrous oxide and 50% oxygen). The woman in labor administered the gas herself through a face mask connected to a valve, which allowed the patient to inhale fresh gas with each breath. Gas administration was continued until the end of the contraction, at which point the patient was breathing room air. Monitoring during labor consisted of a continuous fetal electrocardiogram and tocometer. In addition, the patient’s vital parameters were measured periodically. After approximately one hour of regular uterine contractions, abrupt, deep (up to approximately 50 beats per minute), prolonged deceleration of FHR was observed (Figure 1). The oxytocin infusion was stopped immediately. In total, the patient received approximately 300 mUI of oxytocin. During the examination, she complained of increasing abdominal pain and the abrupt cessation of contractions. Examination revealed a 6-cm dilated cervix with a preserved amniotic bladder. Subsequently, in order to assess amniotic fluid staining, an amniotomy procedure was performed—a large amount of bloody amniotic fluid (about 300–350 mL) was obtained. Vital parameters were stable. Suspecting a uterine rupture, the patient was wheeled to the operating room for an emergency cesarean section under general anesthesia. 

Two 16-gauge intravenous cannulae were immediately secured, and 1000 mL of the multi-electrolyte solution was infused rapidly prior to the induction of anesthesia. Induction was conducted intravenously with propofol and succinylcholine, and rapid tracheal intubation was performed. Anesthesia was maintained with air, oxygen, and 2% (1 MAC) sevoflurane. At the same time, the patient’s blood type was immediately confirmed and 4 units of fresh frozen plasma (FFP), 4 units of red blood cell concentrate (RBC), and 4 units of cryoprecipitate were reserved according to the Holocomb algorithm (the RBC:FFP ratio of 1:1). In addition, the availability of fibrinogen concentrate (hFC) and prothrombin complex concentrate (PCC) has been confirmed as an emergency bridging therapy until blood components are available. 

**Figure 2:**
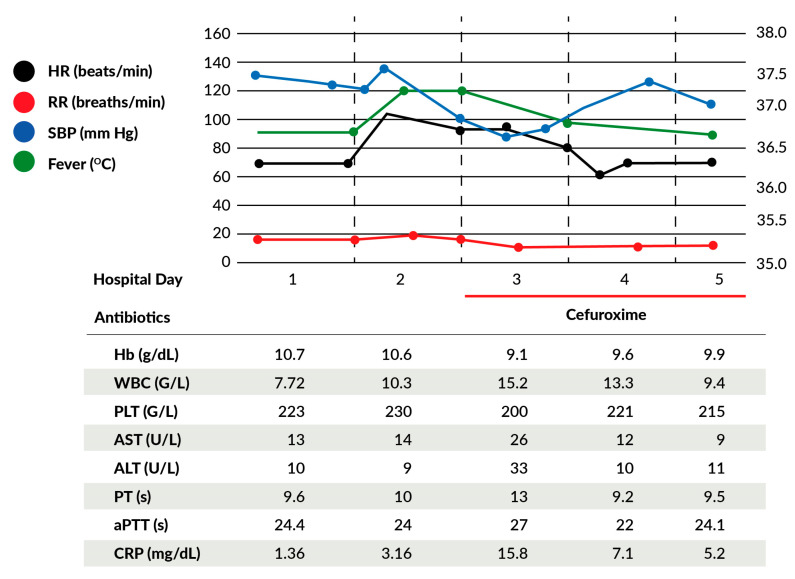
Diagram showing the fluctuations in the patient’s parameters during hospitalization: hemoglobin (Hb), white blood cells (WBC), platelets (PLT), aspartate transaminase (AST), alanine transaminase (ALT), prothrombin time (PT), activated partial thromboplastin time (aPTT), C-reactive protein (CRP).

The abdomen was rapidly opened by a Joel-Cohen incision. A significant amount of bloody amniotic fluid was encountered in the abdominal and pelvic cavities. The previous cesarean section scar was ruptured entirely, and the fetus was partially outside the uterus. The head and shoulders of the fetus were in the abdominal cavity, while the rest of its body remained in the uterine cavity. The fetus was delivered without a fetal heart tone and immediately passed to the neonatal team, who successfully resuscitated the baby after 3 min. The newborn girl of weight 3150 g had an Apgar score of 0/6/8/8 at 1, 3, 5, and 10 min. The uterine rupture was confirmed, with complete damage to the anterior uterine wall from the left to the right broad ligament. One hundred micrograms of Carbetocin and 1 g of tranexamic acid were infused. Blood loss was estimated at 500–600 milliliters (mL). The patient received 1500 mL of multi-electrolyte solution in total. The anatomy of the uterus was not affected—there was no need for an emergency hysterectomy. The rupture was closed with two layers of sutures. Blood and clots were removed. The patient’s pelvis showed no other abnormalities. Further examination showed no damage to the uterine ligaments, adnexes, rectum, or urinary bladder. Vital signs (including Shock Index and mean arterial pressure), hemoglobin, platelet count, clotting times, and lactate levels were within the normal range during and after surgery. One hour after surgery, the patient was extubated in the recovery room and transferred to the maternity ward. The patient was monitored and placed on intravenous cefuroxime and analgesics after surgery. These were continued for four days postoperatively. The patient did not develop any alarming signs and symptoms in the postoperative period. The patient made an uneventful and complete recovery and was discharged home after 4 days with a healthy baby (Figure 2).

## 3. Discussion

Pregnant women with a history of CS are considered a high-risk group because they are likely to develop uterine rupture. Although the risk of uterine rupture is low, its course can be extremely dramatic and lead to the death of the mother and her unborn child. Furthermore, as seen in the presented example, uterine rupture may not only affect women who give birth vaginally after a cesarean section for the first time. It also applies to women who have already passed a successful VBAC.

As mentioned above, a large increase in the cesarean section rate over the past few years can be observed. The frequency of CS nowadays means that most obstetricians will encounter VBACs regularly. TOLAC is one strategy to decrease the rate of cesarean births. TOLAC is associated with less maternal mortality and morbidity than ERCD [12,13]. It should be noted that a successful TOLAC has the fewest complications and is the safest delivery route. In comparison, ERCD can be associated with a risk of placenta accreta in future pregnancies and pelvic adhesions. Factors that contribute most to the uterine rupture are the induction of labor, a shorter interval between deliveries, previous uterine surgeries, macrosomia, abnormal placentation, and prolonged labor. Amongst possible complications associated with TOLAC, uterine rupture is associated with the largest increase in maternal and neonatal morbidity [14,15,16]. Typical uterine rupture symptoms are abdominal pain that begins with a “ripping” sensation, chest pain that may occur if blood enters the peritoneum, heavy vaginal bleeding, and loss of station of the presenting part of the fetus with cessation of uterine contractions [17]. However, the pathological CTG (showing FHR abnormalities) pattern is by far the most frequent (and sometimes the only) symptom [18]. In our case, the pathological CTG record was the first symptom of upcoming events. Although an incorrect CTG pattern may not provide certainty in the diagnosis of a ruptured uterus, its presence should lead to raised awareness of the medical team of a possible uterine rupture, especially during TOLAC [19]. Continuous, uninterrupted CTG monitoring enables the immediate detection of fetal heart rhythm disturbances, which increases the awareness of an ongoing uterine rupture. Therefore, we strongly recommend that continuous CTG monitoring is routinely used in the case of TOLAC. 

It is also noteworthy that labor epidural anesthesia may mask the pain associated with a uterine rupture and lead to a delayed diagnosis [20]. On the other hand, epidural anesthesia is one of the most favorable prognostic factors of a successful TOLAC.

In addition, it is worth considering that the patient had already successfully undergone TOLAC. We bring this to attention as the possible cause of the uterine rupture, in this case, may have been unrecognized uterine muscle incomplete dehiscence during the first VBAC, and this may have contributed to the extensive dehiscence during the subsequent TOLAC. Uterine dehiscence is a condition similar to uterine rupture but characterized by the incomplete division of the uterus that does not penetrate all layers. Such dehiscence can significantly weaken the uterine wall, making it more susceptible to rupture in the next delivery. In addition, such a dehiscence may remain clinically silent and be an incidental finding during a caesarean section [21].

The ultrasonographic evaluation of the lower uterine segment (LUS) can effectively predict the quality of the uterine scar. A lower uterine segment thickness of less than 2 mm is considered a criterion for poor healing and differentiates the risk group of potential uterine rupture with sensitivity and specificity of 86.7% and 100%. In our case, the measurement of the lower section was 3.4 mm [21,22,23]. Additionally, a full LUS thickness of 2.3 mm in women during the first stage of labor is associated with a high risk of complete uterine rupture during a trial of labor. We believe that measuring the full thickness of the LUS may be of practical use when deciding on the mode of delivery and may lead to a reduction in the incidence of uterine ruptures [24]. Despite the high predictive value of this measurement, in our case, it did not protect the patient from uterine rupture. However, considering the above, we believe that LUS measurement should be routinely performed in patients after a cesarean section in order to assess the quality of the cesarean scar and possibly assign the patient to a higher-risk group for uterine rupture during TOLAC.

Uterine rupture may be associated (in the vast majority of cases) with massive postpartum hemorrhage (PPH). Bleeding can be the result of massive damage to the uterine muscle (and surrounding tissues) or the result of atony of the uterine muscle that develops due to rupture. Traditionally, postpartum hemorrhage (PPH) has been defined as greater than 500 mL estimated blood loss associated with vaginal delivery or greater than 1000 mL estimated blood loss associated with cesarean delivery. In our case, no massive bleeding occurred, and the patient did not develop coagulopathy. In addition, the hemoglobin level remained stable throughout. This is consistent with the literature that we have reviewed. Massive hemorrhage is rather associated with the event of uterine rupture, rather than the complete dehiscence of a previous uterine scar. Additionally, in the literature, a greater prevalence of bleeding or the need for transfusions after complete dehiscence of a previous cesarean delivery with associated emergent CS vs. ERCD after the last cesarean delivery is not clearly evident [25,26]. 

Despite the fact that, in our case, there was no development of hemorrhagic shock and coagulopathy, we took all steps to be prepared for such a scenario. Most women die within the first day of the postpartum period, and as many as 88% during the first 4 h of the onset of hemorrhage [27]. Therefore, it is crucial to act rapidly in the case of PPH. Expecting massive bleeding, we secured the essentials of hemorrhage management for immediate action if needed. During the entire course of the cesarean section, we monitored the patient on an ongoing basis for the development of coagulation disorders and increased bleeding. 

Massive hemorrhage requires the immediate action of the entire medical team. Suspecting uterine rupture, we immediately placed the entire team (including anesthesia and operating theater (OR) staff) on alert for a possible ongoing, urgent case. We believe that such a model of action significantly reduces the time needed to react and greatly contributes to improving the quality of care in case of a massive hemorrhage. Furthermore, we believe that proceeding with TOLAC requires the presence of an experienced team (including operating room staff and anesthesiologists) capable of an immediate response in the event of dangerous obstetric events. 

In our case, we applied tranexamic acid (TXA) immediately after starting the operation. We believe that the inhibition of fibrinolysis is essential in the event of severe bleeding. It is of crucial importance to inhibit fibrinolysis via the prompt intravenous administration of tranexamic acid (TXA) in the event of possible PPH. According to the reviewed literature, TXA administration is associated with a significant decrease in blood loss (even up to 50%) and a decrease in the need for blood transfusion [28,29,30,31]. TXA is a safe drug, relatively cheap, and available in most centers. Additionally, recent studies have shown the high safety of a single administration of up to 4 g of TXA in the context of massive bleeding [32,33]. In addition, some guidelines even recommend the routine use of TXA up to 30 min before the planned surgical procedure, to protect the patient in the event of excessive hemorrhage [34,35]. Considering the above, we recommend that TXA should always be administered (along with uterotonics) when postpartum hemorrhage is suspected. In addition, we believe that TXA should be considered before elective cesarean delivery to reduce estimated blood loss.

It is also worth noting that in the case of severe postpartum hemorrhage (sPPH), capillary blood analysis may be helpful in order to assess the intensity of hypovolemic shock [36,37]. Using the critical parameter analyzer, it is possible to quickly assess the acid–base balance (including base excess parameter) and basic parameters such as the hemoglobin level. In addition, such a test may have predictive value in assessing the adaptation of the circulatory system to ongoing hemorrhage. Therefore, we strongly recommend the use of this quick and cheap test to assess the severity of hemorrhage in the presence of ongoing sPPH.

Another catastrophic complication of uterine rupture that can occur is amniotic fluid embolism (AFE) [38,39]. AFE is characterized by a breach of the barrier between the maternal blood and amniotic fluid that forces the entry of amniotic fluid, fetal cells, or other debris into the systemic circulation, causing cardiovascular collapse and acute coagulopathy. AFE is an extremely rare complication—its occurrence is estimated at about 1 in 80,000 deliveries. However, its occurrence is associated with catastrophic consequences and a very low survival rate [39]. The rapid course of AFE and its often irreversible consequences force clinicians to act immediately. According to the latest research, in the case of this dangerous complication, the use of the atropine–ondansetron–ketorolac (AOK) algorithm may prove effective and may improve the patient’s prognosis [40]. We believe that the AOK scheme could be successfully supplemented in the current AFE management guidelines. We raise this topic because we strongly suggest that in the event of such emergencies as a rupture of the uterine muscle, the possibility of AFE should be additionally taken into account.

There are many risk factors for uterine rupture. However, referring to the literature, the dominant risk factor for rupture of the pregnant uterus is a history of previous uterine surgery. The largest percentage of uterine ruptures is caused by surgical treatment of uterine fibroids (especially with the opening of the endometrial cavity). Regardless of the method of myomectomy (hysteroscopy, laparoscopy, or laparotomy), there is a large positive correlation between a history of uterine surgery and uterine rupture. An additional risk factor is the mere presence of large uterine fibroids (with a maximum diameter above 4 cm). Interestingly, the risk of uterine rupture after laparoscopic or abdominal laparotomy is highest in the third trimester of pregnancy. In contrast, the risk of uterine rupture after hysteroscopic myomectomy is more common in the earlier gestational ages. Surprisingly, uterine rupture during pregnancy after abdominal myomectomy seems to be less frequent than after a laparoscopic one [41,42,43]. Taking all the above into consideration, we believe that it is mandatory to consider the eventuality of uterine rupture in pregnancy (particularly in the third trimester) regardless of the type of uterine surgery performed.

Induction of labor with oxytocin infusion is certainly a risk factor for intrapartum uterine rupture. According to the studies conducted, induced labor may increase the risk of uterine rupture during TOLAC. The rate of uterine rupture in women attempting TOLAC with induced labor is slightly higher than with spontaneous labor (2.2% vs. 0.7%) [44]. However, in our case, considering the clinical situation, oxytocin infusion was the procedure of choice. The safest of the recommended procedures was selected using a low-dose oxytocin dosing regimen (in accordance with Polish recommendations). At the same time, we conducted continuous monitoring of the patient and fetus.

Factors associated with a statistically significantly increased likelihood of VBAC are the following: previous VBAC, white race, fetal malpresentation as the indication for a previous cesarean section, estimated fetal weight less than 4000 g, gestation age 37–40 weeks, and spontaneous labor. Although the patient met most criteria for a successful TOLAC, this did not protect her from uterine rupture. Therefore, we believe that no matter how many criteria patients meet, the possibility of uterine rupture should always be considered when attempting a TOLAC [45,46].

Thanks to the raised awareness and the knowledge of the typical symptoms and risk factors of this dangerous event, uterine rupture was our primary diagnosis. Taking quick, decisive action prevented the occurrence of significant fetal complications and prevented the occurrence of a dangerous hemorrhagic shock, which could have led to the patient’s death. 

## 4. Conclusions

Overall, despite its rarity, specifying and pointing to individual cases of uterine rupture should make clinicians aware that the problem of intrapartum uterine rupture is extremely important and still often encountered in everyday practice. A previous successful TOLAC may falsely alleviate the vigilance of the medical staff, which may have disastrous consequences. Therefore, we believe that regardless of the number of previous VBACs, it is crucial to always show extreme vigilance, analyze the risk factors, and conduct constant monitoring of both the mother and the fetus. Thanks to this, even in the event of this dangerous complication, it is possible to take immediate therapeutic action. 

## 5. Clinical Suggestions

Here, we would like to present clinical suggestions on how to manage vaginal delivery after a cesarean section, which are based on the reviewed literature and our personal clinical experience.

- The lower uterine segment should be routinely measured to assess the continuity of the uterine scar. Consider disqualifying a patient from TOLAC if LUS measurement is <2 mm.
- Continuous CTG monitoring should be routinely used in any case of TOLAC.
- Uterine rupture should always be considered in the presence of an abnormal CTG, vaginal bleeding, or abrupt cessation of contractions during TOLAC.
- Regardless of the severity of bleeding in the event of uterine rupture, all components of the hemorrhage management algorithm should always be secured to minimize the time needed to implement them.
- When uterine rupture or heavy bleeding is diagnosed, administer TXA intravenously immediately (along with uterotonics).
- Although a history of VBAC reduces the risk of uterine rupture in a subsequent pregnancy, any patient undergoing IOL should be considered at increased risk for uterine rupture.
- Patients who have undergone previous uterine surgery (regardless of the surgical method) should be treated with extreme caution due to the high risk of uterine rupture during labor.
- The fulfilment of the predictors of successful vaginal delivery after cesarean section should not change the means of monitoring the patient. Such a patient should continue to be treated as a high-risk patient for uterine rupture.
- In the event of uterine rupture, the possibility of AFE should be considered.
- In the event of sPPH, we strongly recommend to use capillary blood test analysis to assess the intensity of hypovolemic shock.
- Proceeding with TOLAC requires the presence of an experienced team (including obstetrician, OR staff, and anesthesiologists) capable of an immediate response in the event of dangerous obstetric events.

## Data Availability

The data presented in this study are available on request from the corresponding author. The data are not publicly available due to privacy restrictions.
